# Saving Resources: SARS-CoV-2 Diagnostics by Real-Time RT-PCR Using Reduced Reaction Volumes

**DOI:** 10.3390/diseases9040084

**Published:** 2021-11-15

**Authors:** Sabine Bock, Bernd Hoffmann, Martin Beer, Kerstin Wernike

**Affiliations:** 1 Berlin-Brandenburg State Laboratory, 15236 Frankfurt, Germany; Sabine.Bock@Landeslabor-bbb.de; 2 Institute of Diagnostic Virology, Friedrich-Loeffler-Institut, 17493 Greifswald, Germany; bernd.hoffmann@fli.de (B.H.); martin.beer@fli.de (M.B.)

**Keywords:** SARS-CoV-2, COVID-19, coronavirus, diagnostics, virus detection, swab, real-time RT-PCR

## Abstract

Since the beginning of 2020, the betacoronavirus SARS-CoV-2 is causing a global pandemic of an acute respiratory disease termed COVID-19. The diagnostics of the novel disease is primarily based on direct virus detection by RT-PCR; however, the availability of test kits may become a major bottleneck, when millions of tests are performed per week. To increase the flexibility of SARS-CoV-2 diagnostics, three real-time RT-PCR assays listed on the homepage of the World Health Organization were selected and investigated regarding their compatibility with three different RT-PCR kits. Furthermore, the reaction volume of the PCR chemistry was reduced up to half of the original protocol to make the individual reactions more cost- and resource-effective. When testing dilution series of culture-grown virus, nearly identical quantification cycle values (Cq) were obtained for all RT-PCR assay/chemistry combinations. Regarding the SARS-CoV-2 detection in clinical samples, agreeing results were obtained for all combinations for virus negative specimens and swabs containing high to medium viral genome loads. In cases of very low SARS-CoV-2 genome loads (Cq > 36), inconsistent results were observed, with some test runs scoring negative and some positive. However, no preference of a specific target within the viral genome (E, RdRp, or N) or of a certain chemistry was seen. In summary, a reduction of the reaction volume and the type of PCR chemistry did not influence the PCR sensitivity.

## 1. Introduction

In late 2019, an outbreak of an acute respiratory disease of humans was reported in Wuhan, China [1]. The novel disease, named COVID-19 (coronavirus disease 2019), very rapidly evolved into a global pandemic [2], resulting in millions of infections in all corners of the world and more than four and a half millions deaths in just under two years of virus circulation [3]. As a causative agent of the pandemic, a betacoronavirus referred to as severe acute respiratory syndrome coronavirus 2 (SARS-CoV-2) was identified [4].

COVID-19 is characterized by mild to moderate respiratory symptoms including pneumonia that can develop into a severe or critical disease, with much higher fatality rates in the elderly or when certain underlying health conditions affecting the cardiovascular, respiratory, or immune systems exist [2]. Nevertheless, also asymptomatic infections occur frequently, and it has been estimated that virus transmission from asymptomatic or pre-symptomatic humans accounts for about half of all COVID-19 cases [5]. This might be in particular critical when asymptomatically infected health care workers transmit the virus into homes for the elderly or hospitals. Onward transmission can be substantially reduced by containment measures such as physical distancing and population-level movement restrictions [6,7,8].

The diagnostics of the novel disease is currently primarily based on the detection of viral antigen by rapid lateral flow tests or, more commonly, on SARS-CoV-2 genome by real-time reverse transcription polymerase chain reaction (RT-PCR) using oropharyngeal or nasopharyngeal swabs as sample material. To identify and isolate infected persons, millions of RT-PCR tests are carried out around the world [9]. Hence, COVID-19 not only overloads national health care systems but also seriously challenges diagnostic capacities when hundreds or thousands of tests need to be performed per day per laboratory [2,9,10]. Although a large number of in-house and commercial real-time RT-PCR assays have been developed and compared regarding their diagnostic performance within a very short time frame [11,12,13,14,15,16], the timely and comprehensive diagnostics might be impaired by supply shortage of the PCR chemistry, as the availability of test kits has become a major bottleneck. Having several widely used assays rely on the identical PCR chemistry [13] might further complicate the wide availability of the necessary reagents. Here, we investigated several real-time RT-PCR assays regarding their combinability with diverse PCR master mixes to increase the flexibility of SARS-CoV-2 PCR diagnostics. In addition, the total reaction volumes were reduced to make the individual reactions more cost- and resource-effective. The selected real-time RT-PCR assays target different genomic regions, further increasing the flexibility, but also the diagnostic specificity, e.g., when contaminations with synthetic control material based on individual viral genes occur, which is a phenomenon that seemed to be widespread in the early stage of COVID-19 diagnostics [17,18]. The SARS-CoV-2 specific real-time PCRs were combined with internal control (IC) assays, which is highly recommended as a quality control measure to avoid false-negative results.

## 2. Materials and Methods

### 2.1. RNA Standard, Virus and Diagnostic Samples

As a PCR standard, purified RNA of SARS-CoV-2 cell culture supernatant was used (stock concentration 1 × 10^4^ copies per µL; provided by Charité—Universitätsmedizin Berlin, Germany, via European Virus Archive goes global (EVAg), Ref-SKU: 026N-03889). A dilution series was prepared in RNA-safe buffer [19], and each dilution step was tested in three replicates. Samples containing genome concentrations near the presumed limit of detection (1 × 10^1^ copies/µL, 2 × 10^0^ copies/µL and 1 × 10^0^ copies/µL) were tested in six replicates as suggested previously [20].

The SARS-CoV-2 virus isolate 2019_nCoV Muc-IMB-1 (kindly provided by R. Woelfel, German Armed Forces Institute of Microbiology, Munich, Germany) was propagated in Vero E6 cells (L1062, collection of cell lines in veterinary medicine, Friedrich-Loeffler-Institut, Greifswald-Insel Riems, Germany). Nucleic acid from different virus preparations was extracted using the QIAamp Viral RNA Mini kit (Qiagen, Hilden, Germany), and all samples were tested in the reactions described below in duplicates.

A total of 93 human pharyngeal swabs originating from different German federal states were submitted for routine diagnostics (legal provisions defined by the German Infection Protection Act (IfSG)). Viral RNA was extracted using the NucleoMag VET kit (MACHERY-NAGEL GmbH & Co. KG, Düren, Germany) according to the supplier’s recommendation.

### 2.2. Real-Time RT-PCRs

The real-time RT-PCR systems “IP4”, “E-Sarbeco”, and “CDC-N3” that are listed on the website of the World Health Organization (WHO) for the genome detection of SARS-CoV-2 [13] were selected and established in our laboratories. To increase the diagnostic accuracy, assays targeting different genomic regions were chosen. The real-time RT-PCR IP4 is based on the RNA-dependent RNA polymerase (RdRp) gene [13], while the assay E-Sarbeco targets the E gene coding region [21], and the assay CDC-N3 targets the nucleocapsid (N) gene [13]. At first, the IP4 assay was established as published in combination with the SuperScript III One Step RT-PCR kit (Thermo Fisher Scientific, Darmstadt, Germany; 882 € per 100 reactions) using the previously described reaction conditions [13] and 5 µL RNA template. Thereafter, the reactions were modified by replacing the PCR chemistry by (1) the AgPath-ID One-Step RT-PCR Reagents kit (Thermo Fisher Scientific; 255 € per 100 reactions) or (2) the qScript XLT One-Step RT-qPCR ToughMix kit (Quantabio, Beverly, MA, USA; 211 € per 100 reactions). For a single reaction, the following components were merged to a master mixture: (1) 4.5 µL RNase-free water, 12.5 µL 2 x RT-PCR Buffer, 1 µL 25 x RT-PCR enzyme mix, and 2 µL of the respective primer/probe mix (600 nM for each primer and 200 nM probe); (2) 5.5 µL RNase-free water, 12.5 µL 2 x qScript XLT One-Step RT-qPCR ToughMix and 2 µL of the respective primer/probe mix (600 nM for each primer and 200 nM probe). In both cases, 5 µL of RNA template was added. In a next step, the volume of the master mixture was reduced, resulting in 10 µL for a single reaction, and either 5 µL or 2.5 µL of RNA template was added. The real-time RT-PCRs were carried out using the thermal profiles listed in Table 1. The limit of detection (LOD) was calculated by using a web service funded by BVL (Berlin, Germany; https://quodata.de/content/validation-qualitative-pcr-methods-single-laboratory (accessed on 2 November 2021)).

All the following tests were performed in a reduced total reaction volume and in comparison to the assay IP4.

As a next step, a dilution series of RNA extracted from a whole virus preparation was tested by each of the three SARS-CoV-2 PCRs, i.e., IP4, E-Sarbeco and CDC-N3, in combination with the AgPath-ID One-Step RT-PCR Reagents kit (Thermo Fisher Scientific) or the qScript XLT One-Step RT-qPCR ToughMix kit (Quantabio, Beverly, MA, USA). To further increase the flexibility of the real-time RT-PCR setup, a third PCR kit was included, namely the Luna^®^ Universal Probe One-Step RT-qPCR kit (New England Biolabs GmbH, Frankfurt am Main, Germany; 198 € per 200 reactions). For a single reaction, 4.5 µL RNase-free water, 5 µL 2 × Luna Universal Probe One-Step Reaction Mix, 0.5 µL 20 × Luna WarmStart^®^ RT Enzyme Mix, and 2.5 µL RNA template were merged. For amplification, the reaction was carried out at the temperature profile given in Table 1.

Finally, the SARS-CoV-2 real-time RT-PCR assays were combined with an IC system based on the housekeeping gene beta-actin [22] or with a heterologous IC [19] to control for efficient RNA extraction and amplification, thereby preventing false-negative results. The duplex PCRs were exemplarily tested in combination with the AgPath-ID One-Step RT-PCR Reagents kit (Thermo Fisher Scientific) in a total reaction volume of 12.5 µL.

In order to evaluate the diagnostic sensitivity and specificity of the modified real-time RT-PCRs, the clinical swab samples were investigated by each of the three SARS-CoV-2 RT-PCRs in combination with the ICs and either the AgPath-ID One-Step RT-PCR Reagents kit (Thermo Fisher Scientific) or the qScript XLT One-Step RT-qPCR ToughMix kit (Quantabio). The diagnostic sensitivity, specificity, and accuracy were calculated by using the free statistical calculator MedCalc (MedCalc Software, Ostend, Belgium).

All oligonucleotides were synthesized by either metabion international AG (Planegg, Germany) or TIBMOLBIOL (Berlin, Germany) and the real-time RT-PCR reactions were carried out using a Bio-Rad CFX 96 Real-Time Detection System (Bio-Rad, Hercules, CA, USA).

## 3. Results

### 3.1. Volume Reduction of Reagents to 12.5 µL and Type of Used PCR Chemistry Did Not Influence the PCR Sensitivity

**Figure 1:**
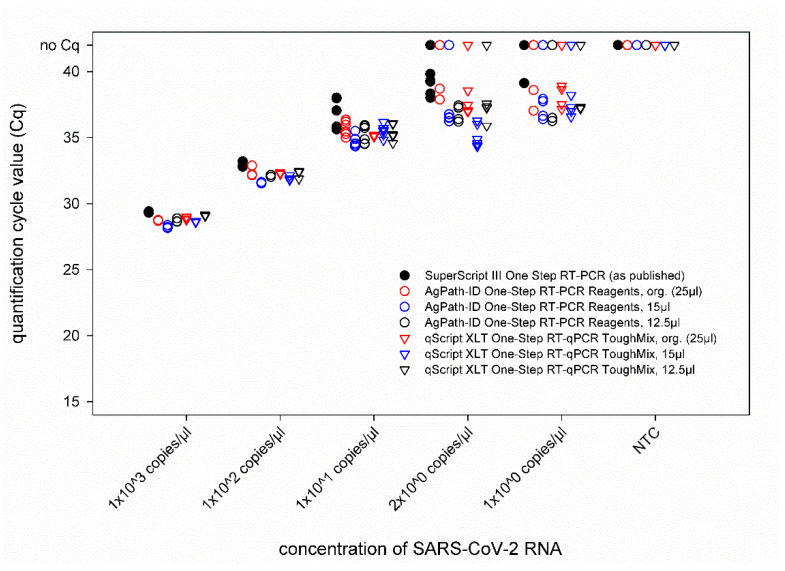
Comparison of the IP4 real-time RT-PCR performed with different real-time RT-PCR kits and total reaction volumes. The newly established protocols were compared to the previously published version of this PCR using SARS-CoV-2 standard RNA. Org.—original protocol described by the manufacturers of the RT-PCR kits. NTC—no template control (=RNase free water).

A dilution series of the RNA standard was tested by the IP4 real-time RT-PCR combined with different volumes of the AgPath-ID One-Step RT-PCR Reagents and qScript XLT One-Step RT-qPCR ToughMix kits in comparison to the published protocol [13]. From 10^3^ to 10^1^ copies/µL, every replicate of each approach tested positive, and comparable quantification cycle (Cq) values were obtained (Figure 1). The LOD95% was below 10 for every approach (Supplementary Table S1). For SARS-CoV-2 dilutions containing 2 × 10^0^ or 1 × 10^0^ copies/µL, some of the replicates scored negative. However, an influence of a specific PCR kit or total reaction volume could not be observed, as none of the approaches failed to detect every replicate of a given RNA concentration, and even these very low concentrations could be detected by RT-PCR set-ups using reduced volumes (Figure 1). Hence, all the following analyses were carried out using a total reaction volume of 12.5 µL.

**Figure 2:**
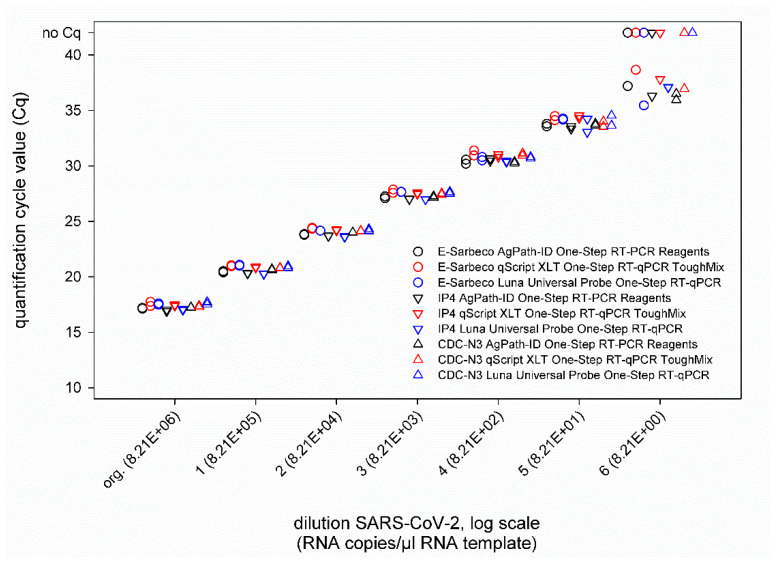
Results of the SARS-CoV-2 real-time RT-PCRs E-Sarbeco, IP4 and CDC-N3 performed in combination with either the AgPath-ID One-Step RT-PCR Reagents kit (black), the qScript XLT One-Step RT-qPCR kit (red), or the Luna Universal Probe One-Step RT-qPCR kit (blue). All samples were tested in duplicate.

Next, a ten-fold dilution series of culture-grown virus was tested. This virus preparation contained 8.21 × 10^6^ RNA copies per µL RNA template, as was determined based on an external standard. When comparing the three different SARS-CoV-2 real-time RT-PCRs, i.e., IP4, E-Sarbeco, and CDC-N3, run in combination with either the AgPath-ID One-Step RT-PCR Reagents kit, the qScript XLT One-Step RT-qPCR ToughMix kit, or the Luna^®^ Universal Probe One-Step RT-qPCR kit, nearly identical Cq values were obtained in every approach (Figure 2). Only in the highest virus dilution was a certain degree of variability observed, but without a prominent effect of a given real-time PCR chemistry, since in every combination, at least one of the duplicates tested weakly positive (Figure 2).

**Figure 3:**
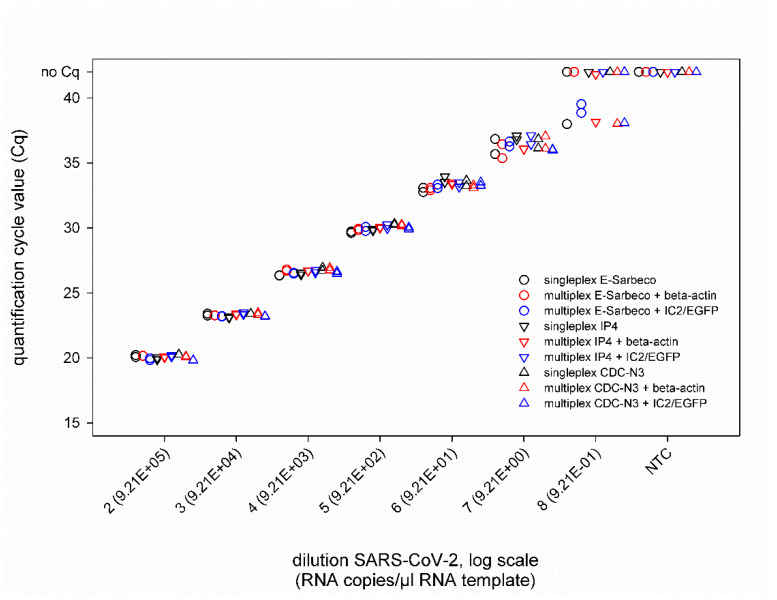
Results of the SARS-CoV-2 real-time RT-PCRs E-Sarbeco, IP4 and CDC-N3 performed either individually (black) or in a duplex reaction with an internal control assay based on the housekeeping gene beta-actin (red) or a heterologous control (IC2/EGFP, blue). All samples were tested in duplicate. NTC—no template control (=RNase free water).

In addition, the combination of the SARS-CoV-2 real-time RT-PCRs with IC systems did not negatively influence the PCR performance (Figure 2), as nearly identical Cq values were measured for SARS-CoV-2 dilutions (second virus preparation) ranging from 10^−2^ to 10^−7^ (corresponding to 9.21 × 10^5^ to 9.21 × 10^0^ RNA copies per µL RNA template) in singleplex and duplex approaches, independent of the applied IC assay (Figure 3). The LOD95% of every approach is given in Supplementary Table S2.

### 3.2. Diagnostic Performance of the Optimized Real-Time PCR Protocols with Clinical Samples

The performance of all duplex real-time RT-PCRs was also investigated using human pharyngeal swabs with two different PCR kits. While the IC systems tested correctly positive in every approach where the duplex PCRs were combined with the AgPath-ID One-Step RT-PCR Reagent kit, the beta-actin based IC scored negative for five or three of the 73 negative swab samples, respectively, when the E-Sarbeco and CDC-N3 PCRs were combined with the qScript XLT One-Step RT-qPCR kit (Table 2). Regarding the SARS-CoV-2 real-time RT-PCRs, agreeing results were obtained for all RT-PCR/chemistry-combinations for virus negative clinical specimens and swabs containing high to medium viral genome loads. In cases of very low SARS-CoV-2 genome loads (Cq > 36), inconsistent results were observed, with some approaches scoring negative and some positive. However, no preference of a specific target within the viral genome (E, RdRp or N) or of a certain PCR chemistry could be observed (Table 2).

## 4. Discussion

Since SARS-CoV-2 became a pandemic in early 2020, this virus is keeping the world in suspense as the control measures affect all areas of life, and the disease itself heavily challenges health care systems and diagnostic capacities. To identify and isolate infected persons, inconceivably large numbers of RT-PCR tests need to be performed [2,9,10]. In comparison to spring and summer (the so-called “first wave”), the number of tests even increased in the northern hemisphere’s autumn 2020 and winter 2020/21, when many countries have faced a “second wave”, i.e., strongly increasing case numbers [23,24]. Furthermore, since mid-2021, multiple countries are experiencing a massive wave of infections driven by the Delta variant or other variants of concerns [25]. In times of growing demand for testing, the availability of test kits may be a problematic issue. To overcome supply shortages of individual reagents, a reduction of the required reaction volume, pooling of samples, or falling back on alternative reagents might be problem-solving approaches. The concept of volume reduction of the applied PCR chemistry has been already proposed and successfully implemented recently for diagnostics of influenza A viruses in low- and middle-income countries [26]. Here, it could be demonstrated that a significant reduction of PCR reagents to 12.5 µL/well without negatively influencing the sensitivity of SARS-CoV-2 RT-PCR assays is likewise possible. In addition, an adequate robustness of the applied RT-PCR protocols was observed, even when diverse master mixes were used, further increasing the flexibility in times of overwhelming need for SARS-CoV-2 PCR diagnostics. However, when new assay/chemistry combinations not included in this comparison are to be used, they should be validated in comparison to well-established systems, since the PCR chemistry could negatively influence the performance of the test. This was e.g., shown in the present study for the IC that failed to react correctly positive in very few cases when two of the duplex PCR systems were combined with the qScript XLT One-Step RT-qPCR kit.

An additional option to save resources when dealing with large amounts of sample specimens is pooling approaches, since they could drastically increase the sample throughput. In veterinary medicine, this experience was made with a variety of pathogens, among them numerous viruses and diverse sample materials already years ago [26,27,28,29], and only recently, several groups confirmed the applicability of sample pooling for SARS-CoV-2 diagnostic as well [30,31,32,33,34]. However, pooling is only a useful option in situations of a low virus prevalence, e.g., in screening asymptomatic populations in regions with only low-level virus circulation, as every positive pool needs to be resolved to test each included sample individually. When pooling of samples is further combined with reduced PCR volumes, which can be ideally used flexibly with different PCR master mixes, the diagnostic capacity could be drastically increased. Such approaches are in particular beneficial when health care workers can be regularly screened for infection even in resource-limited settings, thereby avoiding the transmission of SARS-CoV-2 into care homes of the elderly, which are most at risk of severe disease and death when the residents are not yet vaccinated [35,36].

Within the different duplex RT-PCR protocols established in the present study, the tested SARS-CoV-2 assays proved to be equally sensitive as the corresponding single-target RT-PCRs, which excludes a negative effect of the IC amplification, while the inclusion of ICs markedly increases the diagnostic certainty. However, in cases of very low SARS-CoV-2 genome loads, inconsistent results were observed in the virus-specific RT-PCRs. Such low viral RNA loads, which are near the limit of detection of the diagnostic systems, can be found in the upper respiratory tract in later phases after infection [37,38,39] and most likely do not equate to infectiousness [40,41]. In a previous study on COVID-19 patients, it was shown that the probability of isolating infectious virus was less than 5% when the viral RNA load was below 6.63 Log10 RNA copies/mL [42]. This is strikingly similar to the cut off of 6.51 Log10 RNA copies/mL reported in another study [39]. These observations and values implicate that patients below this value are not infectious anymore.

Nevertheless, the re-testing of the person concerned is highly recommended, to exclude, e.g., suboptimal sampling.

## 5. Conclusions

In the current comparative study, neither a marked reduction of the total reaction volume nor the type of RT-PCR chemistry negatively influenced the sensitivity of the applied real-time RT-PCR assays, as shown by equivalent results when testing dilution series of SARS-CoV-2 standard RNA or RNA extracted from culture-grown virus. However, when other RT-PCR kits that were not included in this study are to be used for SARS-CoV-2 diagnostics, an initial validation of the new kit and of the reaction conditions (e.g., volume, thermal profile) is highly recommended. For this, one should compare the intended protocol to a well-validated system, preferably the originally published protocol.

Since inconsistent results were observed in cases of very low SARS-CoV-2 genome loads (Cq > 36, <100 genome copies) in this study, two independent RT-PCR assays should be applied in all cases, ideally targeting different genomic regions, for the reliable diagnostics of SARS-CoV-2 infections by real-time RT-PCR even in patients with very low genome loads at the time of sampling.

## Figures and Tables

**Table 1:** Thermal profiles of the RT-PCR chemistry used in this study.

Reaction Step	SuperScript III One Step RT-PCR Kit	AgPath-ID One-Step RT-PCR Reagents Kit	qScript XLT One-Step RT-qPCR ToughMix Kit	Luna^®^ Universal Probe One-Step RT-qPCR Kit
reverse transcription	55 °C for 20 min	45 °C for 10 min	50 °C for 10 min	55 °C for 10 min
PCR initial activation	95 °C for 3 min	95 °C for 10 min	95 °C for 1 min	95 °C for 1 min
three step cycling (42 cycles):				
denaturation	95 °C for 15 s	95 °C for 15 s	95 °C for 15 s	95 °C for 10 s
annealing	58 °C for 30 s	57 °C for 20 s	57 °C for 20 s	57 °C for 20 s
extension	(combined with annealing)	72 °C for 20 s	68 °C for 30 s	68 °C for 30 s

**Table 2:** Clinical performance of the SARS-CoV-2 real-time RT-PCRs that were optimized for a low total reaction volume. Seventy-three negative and 20 presumably SARS-CoV-2 positive human pharyngeal swabs were investigated using three different real-time RT-PCRs combined with an internal control based either on the housekeeping beta-actin gene or a heterologous control RNA (=IC2/EGFG). The first values were generated by combining the indicated real-time RT-PCR with the AgPath-ID One-Step RT-PCR Reagents kit, while the second values were produced with the qScript XLT One-Step RT-qPCR ToughMix kit. Disagreeing results are indicated in bold. neg.—negative, ^1^ 68 out of 73 samples tested positive in the internal control assay, ^2^ 70 out of 73 samples tested positive in the internal control assay.

Sample Material	E-Sarbeco				IP4				CDC-N3			
	SARS-CoV-2	Beta-Actin	SARS-CoV-2	IC2/EGFP	SARS-CoV-2	Beta-Actin	SARS-CoV-2	IC2/EGFP	SARS-CoV-2	Beta-Actin	SARS-CoV-2	IC2/EGFP
human pharyngeal swab (n = 73)	neg.	28.8 ± 3.0/ 31.3 ± 4.9 ^1^	neg.	23.9 ± 0.1/ 24.6 ± 0.2	neg.	29.7 ± 3.1/ 30.5 ± 2.8	neg.	24.4 ± 0.2/ 24.8 ± 0.1	neg.	28.5 ± 3.1/ 29.9 ± 3.4 ^2^	neg.	24.6 ± 0.2/ 23.8 ± 0.2
human pharyngeal swab #74	23.6/23.8	28.3/29.4	24.3/24.0	25.6/26.2	23.9/24.1	28.9/31.4	24.4/24.2	26.0/27.1	24.4/23.5	28.1/29.4	24.2/23.5	25.3/24.3
human pharyngeal swab #75	33.9/34.4	29.4/30.8	35.6/34.5	25.7/25.9	35.0/36.0	29.6/31.5	34.0/33.8	25.7/26.7	35.7/33.1	29.2/30.1	34.9/33.5	25.1/24.1
human pharyngeal swab #76	27.5/28.1	27.2/28.9	28.4/28.1	25.7/26.1	27.8/28.0	27.5/29.5	28.2/28.0	25.5/26.6	28.5/27.3	27.1/28.2	28.4/27.6	24.8/24.1
human pharyngeal swab #77	25.1/25.1	34.4/36.9	25.4/25.2	25.7/26.2	24.7/24.2	34.1/36.0	25.0/25.1	25.7/27.0	25.3/24.2	35.2/neg.	25.1/24.4	24.8/24.1
human pharyngeal swab #78	24.2/24.0	29.0/30.4	25.0/24.3	25.7/26.2	24.1/24.1	29.6/31.4	24.3/24.2	25.5/26.7	24.8/23.8	28.2/29.8	24.5/24.0	24.8/24.1
human pharyngeal swab #79	**neg./neg.**	32.5/34.1	**neg./37.1**	25.7/26.1	**neg./neg.**	32.5/35.1	**neg./neg.**	25.5/26.5	**neg./neg.**	31.5/35.4	**neg./neg.**	24.8/24.1
human pharyngeal swab #80	**neg./37.3**	32.0/34.4	**neg./neg.**	25.4/26.2	**neg./neg.**	32.6/34.9	**neg./37.4**	25.4/26.5	**36.6/neg.**	32.2/34.4	**neg./neg.**	25.0/23.9
human pharyngeal swab #81	26.2/26.1	30.4/31.8	26.8/26.3	25.6/26.1	26.3/26.2	31.2/32.8	26.3/26.3	25.3/26.6	26.7/25.6	30.1/31.8	26.4/25.5	24.8/23.7
human pharyngeal swab #82	28.2/28.7	33.4/35.6	28.2/28.8	25.3/26.1	28.9/28.9	34.4/35.6	28.6/28.8	25.5/26.6	28.5/27.6	33.3/neg.	28.3/27.2	25.3/24.1
human pharyngeal swab #83	29.4/29.4	31.3/33.1	29.9/29.6	25.8/26.3	29.3/29.5	31.7/33.7	29.4/29.4	26.0/26.9	30.2/28.6	30.9/32.8	29.5/28.5	25.2/24.1
human pharyngeal swab #84	**neg./36.2**	32.9/35.3	**neg./neg.**	25.8/26.2	**37.0/37.2**	33.7/35.5	**neg./neg.**	25.9/26.5	**neg./neg.**	33.8/neg.	**36.59/neg.**	24.8/24.0
human pharyngeal swab #85	31.5/31.1	31.7/34.4	31.8/31.9	25.8/26.1	31.4/31.5	32.5/34.3	31.4/31.5	25.6/26.7	31.8/30.7	31.5/33.0	31.4/30.4	24.8/24.1
human pharyngeal swab #86	31.7/31.7	30.1/31.2	32.0/31.6	25.7/26.2	31.7/21.1	29.9/31.7	31.3/31.7	25.6/26.8	31.5/30.2	29.3/30.5	31.7/30.4	24.9/24.1
human pharyngeal swab #87	36.8/36.1	32.7/34.6	34.7/35.5	25.6/26.0	35.0/35.1	33.4/35.1	35.7/35.0	25.6/26.3	38.1/35.8	32.6/34.5	35.0/neg.	24.8/23.8
human pharyngeal swab #88	29.3/29.3	30.3/31.7	29.6/29.6	25.5/25.9	29.4/29.3	31.0/32.9	29.4/29.3	25.5/26.5	30.1/28.3	30.2/31.3	29.8/28.3	24.8/23.7
human pharyngeal swab #89	30.1/31.1	34.2/38.8	31.3/31.1	25.6/26.3	31.7/31.3	34.1/36.4	30.8/31.1	25.1/26.6	31.2/30.1	33.8/neg.	30.7/29.5	24.8/23.8
human pharyngeal swab #90	26.0/26.2	28.8/30.0	26.4/26.2	25.7/26.0	26.1/26.2	29.2/31.3	26.2/26.4	25.6/26.6	26.6/25.6	28.6/27.8	26.5/25.6	25.0/24.2
human pharyngeal swab #91	30.1/30.3	28.0/29.3	30.5/30.1	26.0/26.2	30.2/30.3	28.0/30.1	30.1/30.1	25.6/26.8	30.0/28.7	27.3/28.4	29.7/28.5	24.8/24.2
human pharyngeal swab #92	**36.3/36.0**	28.2/30.0	**neg./36.6**	25.9/26.0	**36.8/38.0**	28.4/30.3	**neg./36.1**	25.4/26.5	**37.8/41.3**	27.7/28.9	**neg./36.1**	24.7/24.0
human pharyngeal swab #93	27.5/27.4	32.1/34.1	27.9/27.5	25.7/26.1	27.2/27.3	32.9/34.8	27.3/27.3	25.4/26.5	28.0/27.0	32.0/34.6	27.5/27.0	24.9/24.0
diagnostic specificity	100.00%/ 100.00%		100.00%/ 100.00%		100.00%/ 100.00%		100.00%/ 100.00%		100.00%/ 100.00%		100.00%/ 100.00%	
diagnostic sensitivity	85.00%/ 95.00%		80.00%/ 90.00%		90.00%/ 90.00%		80.00%/ 90.00%		90.00%/ 90.00%		80.00%/ 95.00%	
accuracy	96.77%/ 98.92%		95.70%/ 97.85%		97.85%/ 97.85%		95.70%/ 97.85		97.85%/ 97.85%		95.70%/ 98.92%	

## Data Availability

The data presented in this study are available in the article.

## Supplementary Materials

The following are available online at https://www.mdpi.com/article/10.3390/diseases9040084/s1, Table S1: Limit of detection LOD95% of the protocols used in this study as determined based on a PCR standard provided by Charité—Universitätsmedizin Berlin, Germany, via European Virus Archive goes global (EVAg), Ref-SKU: 026N-03889. Org.—original protocol. Table S2: Limit of detection LOD95% of the protocols used in this study as determined based on two distinct preparations of culture-grown SARS-CoV-2.
