# Supplementary material for: Saving Resources: SARS-CoV-2 Diagnostics by Real-Time RT-PCR Using Reduced Reaction Volumes
Source: Diseases. 2021 Nov 15;9(4):84. doi: 10.3390/diseases9040084 (PMC8628695; doi:10.3390/diseases9040084)
Supplement: Supplementary file 1 [file diseases-09-00084-s001.zip › Supplementary Table S1.pdf]

Supplementary Table S1. Limit of detection LOD95% of the protocols used in this study as determined based on a PCR standard provided by Charité – Universitätsmedizin Berlin, Germany, via European Virus Archive goes global (EVAg), Ref-SKU: 026N-03889. Org. – original protocol

PCR chemistry/total reaction volume	LOD95%	95% confidence interval
SuperScript III One Step RT-PCR/org. (25 µl, as published)	5.637	2.669, 11.970
AgPath-ID One-Step RT-PCR Reagents/org. (25 µl)	9.219	4.392, 19.422
AgPath-ID One-Step RT-PCR Reagents, 15 µl	3.322	1.600, 6.884
AgPath-ID One-Step RT-PCR Reagents, 12.5 µl	3.440	1.657, 7.126
qScript XLT One-Step RT-qPCR ToughMix/org. (25 µl)	4.030	1.929, 8.412
qScript XLT One-Step RT-qPCR ToughMix, 15 µl	2.101	1.000, 4.453
qScript XLT One-Step RT-qPCR ToughMix, 12.5 µl	4.599	2.184, 9.660
