# Supplementary material for: Saving Resources: SARS-CoV-2 Diagnostics by Real-Time RT-PCR Using Reduced Reaction Volumes
Source: Diseases. 2021 Nov 15;9(4):84. doi: 10.3390/diseases9040084 (PMC8628695; doi:10.3390/diseases9040084)
Supplement: Supplementary file 1 [file diseases-09-00084-s001.zip › Supplementary Table S2.pdf]

Supplementary Table S2. Limit of detection LOD95% of the protocols used in this study as determined based on two distinct preparations of culture-grown SARS-CoV-2.

PCR chemistry/total reaction volume	LOD95%	95% confidence interval
virus preparation 1		
E-Sarbeco, AgPath-ID One-Step RT-PCR Reagents	35.046	8.215, 236.619
E-Sarbeco, qScript XLT One-Step RT-qPCR	35.046	8.215, 236.619
E-Sarbeco, Luna Universal Probe One-Step RT-qPCR	35.046	8.215, 236.619
IP4, AgPath-ID One-Step RT-PCR Reagents	35.046	8.215, 236.619
IP4, qScript XLT One-Step RT-qPCR	35.046	8.215, 236.619
IP4, Luna Universal Probe One-Step RT-qPCR	6.705	0.839, 53.744
CDC-N3, AgPath-ID One-Step RT-PCR Reagents	6.705	0.839, 53.744
CDC-N3, qScript XLT One-Step RT-qPCR	35.046	8.215, 236.619
CDC-N3, Luna Universal Probe One-Step RT-qPCR	102.628	21.628, 492.456
virus preparation 2		
singleplex E-Sarbeco	3.929	0.921, 26.529
multiplex E-Sarbeco + beta-actin	11.506	2.425, 55.212
multiplex E-Sarbeco + IC2/EGFP	1.151	0.242, 5.458
singleplex IP4	11.506	2.425, 55.212
multiplex IP4 + beta-actin	3.929	0.921, 26.529
multiplex IP4 + IC2/EGFP	11.506	2.425, 55.212
singleplex CDC-N3	11.506	2.425, 55.212
multiplex CDC-N3 + beta-actin	3.929	0.921, 26.529
multiplex CDC-N3 + IC2/EGFP	3.929	0.921, 26.529
